# Effects of Plantation Type and Soil Depth on Microbial Community Structure and Nutrient Cycling Function

**DOI:** 10.3389/fmicb.2022.846468

**Published:** 2022-05-31

**Authors:** Wenbo Wang, Jianjun Wang, Qianchun Wang, Ramon Santos Bermudez, Shihe Yu, Pengtu Bu, Zhanwei Wang, Dongshen Chen, Jian Feng

**Affiliations:** ^1^School of Biological Science and Technology, University of Jinan, Jinan, China; ^2^Liaoning Academy of Forestry Sciences, Shenyang, China; ^3^Faculty of Agricultural Sciences, Luis Vargas Torres de Esmeraldas University of Technology, Esmeraldas, Ecuador; ^4^Research Institute of Forestry, Chinese Academy of Forestry, Beijing, China

**Keywords:** mixed plantation, nutrient cycling function, soil microbial community, soil depth, enzyme activity

## Abstract

Declining soil quality and microecological imbalances were evaluated in larch plantations in this study. One potential solution to this problem is the cultivation of mixed coniferous and broad-leaved plantations. However, it is unclear whether and how soil microbial community structure and nutrient cycling function would be affected by mixed plantations and soil depths. In this study, we used high-throughput sequencing technology to investigate bacterial 16S and fungal ITS regions for comparisons of soil microbial diversity among plantation types (a *Larix gmelinii* pure plantation, a *Fraxinus mandshurica* pure plantation, a *Larix*–*Fraxinus* mixed plantation within the *Larix* row, the *Fraxinus* row, and between the *Larix* and *Fraxinus* rows) and soil depths (0–10, 10–20, and 20–40 cm). These data were used to evaluate variations in microbial communities and nutrient cycling function with the determining environmental factors. Our results indicated that bacteria had a stronger spatial dependence than did fungi, while plantation types significantly affected the fungal community. The relative abundance of *Gaiellaceae*, as well as bacterial ligninolysis, nitrate ammonification, and nitrite ammonification functions significantly increased with increasing soil depth. Compared with other plantations, the relative abundance of *Inocybaceae* was significantly higher in the *Larix* plantation. Distance-based redundancy analysis (db-RDA) showed that *Gaiellaceae* and *Inocybaceae* abundances were positively correlated with ammonium nitrogen content, available phosphorus content, and phosphatase activity. Our findings indicate that variations in soil available phosphorus are closely related to the relative abundances of *Gaiellaceae* at different soil depths and *Inocybaceae* in different plantation types. Mixed plantations might change the availability of soil phosphorus by controlling the relative abundance of *Inocybaceae*. We recommend that fungal community changes be considered in the sustainable management of mixed plantations.

## Introduction

The long-term management of pure coniferous plantations has resulted in poor nutrient turnover efficiency, low bio-diversity, and a poor soil ecosystem (Liu et al., 2018). It has been reported that cultivating mixed coniferous and broad-leaved plantations could change stand biomass (Wang et al., 2008), soil microbial community catabolic diversity (Jiang et al., 2012), soil carbon storage (He et al., 2013), litter decomposition, and nutrient return rate (Wang et al., 2007, 2019). Despite some studies reporting on soil bacterial and fungal community composition in different plantation types, there were few studies on the differences of microbial community structure and nutrient cycling function among different tree species belt of mixed plantation. And it remains poorly understood how mixed plantation and soil depth influence specific microbial groups relevant to soil nutrient cycling.

As essential participants in the nutrient cycling and energy flow, soil microbial communities have important roles in reestablishing function and biodiversity of ecosystem restoration (Harris, 2009; Jiang et al., 2012). Furthermore, changes in soil microbial community composition and function can sensitively reflect soil environmental quality changes, an important component of soil productivity (Vasconcellos et al., 2013). The soil nutrient content and enzyme activities are reportedly higher in mixed plantations than in pure plantations (Li et al., 2015). Soil microbial abundance, catabolic potentials, and versatility are significantly influenced by forest vegetation (Garau et al., 2019). Previous studies find that compared with a pure coniferous plantation, mixed coniferous and broad-leaved plantations have been shown to significantly increase soil microbial Shannon diversity, OTU richness, and biological functions involved in carbon (C) and nitrogen (N) cycles (i.e., enzyme activities, carbon utilization patterns, and functional gene abundance; Jiang et al., 2012; Pereira et al., 2018). The study showed that the dominant microbial flora and C, N cycling functional groups in different plantation types of soil were diverse. These differences in microbial composition led to soil acidification and a large decline in available soil nutrient (Zhang et al., 2016). It is critical to study the relative contribution of specific microbial groups to nutrient content and enzyme activities in mixed broad-leaved plantations to understanding the potential microbial mechanism involved with soil nutrient cycling and maintain soil fertility of plantation.

*Larix* is one of the main afforestation tree species in China. Pure larch plantations have some problems, such as soil degradation and acidification (Yang et al., 2010). To improve the ecological stability and soil environment of pure larch plantations, some scholars have investigated combinations of *Larix* with other broad-leaved tree species. The results have shown that mixed plantations are superior to pure plantations (Liu et al., 1998; Wang et al., 2019). Furthermore, the catabolic diversity and function of soil microbial communities are much better under broadleaf tree species and mixed broadleaf–conifer plant species than under coniferous tree species (Jiang et al., 2012). *Fraxinus mandshurica* is an important native broad-leaved tree species in the Eastern Liaoning Province, China. To our knowledge, there are few reports regarding mixed plantations of *Larix* and *Fraxinus* in this area.

The distributions of soil bacteria and fungi are significantly affected by soil depths (Zhang et al., 2016; Sun et al., 2018). In 0–40-cm soil layer, gram-positive bacteria and actinomycetes reportedly exhibited increased abundance with increasing soil depth (Li et al., 2017). In contrast, Gram-negative bacteria and fungi are most abundant at the soil surface and demonstrate substantially lower abundances in subsurface soil layers (Fierer et al., 2003). Although some studies have demonstrated changes in soil microbial communities among forest types and soil depths, little is known regarding the relationships of specific microbial groups with soil nutrient contents in mixed plantations. Hence, there is a need for a thorough understanding of microbial distributions among plantation types and soil depths, as well as complex linkages among microbial consortia, soil characteristics, and environmental conditions; such information may provide novel insights that support the improvement of *Larix* plantation management practices.

Here, we selected a pure *Larix* plantation, a pure *F. mandshurica* plantation, and a mixed *Larix* and *Fraxinus* plantation to compare soil nutrient content, enzyme activity, microbial community composition, and nutrient cycling functional characteristics in five inter-forest zones among three plantation types. This study was performed to explore the structural and nutrient cycling functional characteristics of bacterial and fungal communities among plantation types and soil depths; it was also performed to clarify the relationships among soil microbial communities, enzyme activities, and nutrient contents according to forest type and soil depth. The results of this study will provide a theoretical basis for the scientific evaluation of land productivity in mixed plantations. We hypothesized that responses to plantation types and soil depths would have different effects on bacterial and fungal communities; moreover, we presumed that mixed plantations would influence the abundances of specific microbial groups, leading to alterations in soil nutrient contents.

## Materials and Methods

### Study Sites and Sample Collection

The research site was located in the Douling forest farm in the south-central part of Xinbin Manchu Autonomous County, Liaoning Province, China (geographical coordinates: 124° 52.37′E and 41° 30.41’N). This site is in the Longgang mountain range extension of the Changbai Mountain system. The mean altitude of the territory is approximately 500 m, the mountain slopes are gentle (between 10° and 25°), and the mountains and hills are low. The study area falls within the north temperate seasonal continental climate with four distinct seasons, a pleasant climate, and abundant rainfall (mean annual rainfall of 750–850 mm). The main tree species are *Larix gmelinii*, *Pinus sylvestris* var. *mongolica* Litv., *Pinus thunbergii* Parl, *Pinus koraiensis* Sieb.et Zucc., *Quercus mongolica* Fisch. ex Ledeb., *Juglans mandshurica* Maxim., and *F. mandshurica* Rupr. Shrubs in the plantations mainly include *Euonymus alatus* (Thunb.) Sieb, *Lonicera japonica* Thunb., *Corylus* L., *Aralia elata* (Miq.) Seem., and *Acanthopanax senticosus* (Rupr. et Maxim.) Harms.

Three plantation types of pure *L. gmelinii*, pure *F. mandshurica*, and mixed *L. gmelinii* and *F. mandshurica* were selected in August 2019 as the experimental plots for this study. The selected plots had similar slope aspects, stand age, and stand density. The mixed *Larix* and *Fraxinus* plantation was cultivated in mixed row mode (5 × 5). Three 20 m × 30 m standard plots were established for each forest type using the typical plot method, with a total of nine completely independent and randomly distributed sample plots (Table 1). Samples were randomly collected from 10 points at each sample plot. Soil samples were acquired at depths of 0–10, 10–20, and 20–40 cm in each sample plot. Additionally, in the mixed *Larix* and *Fraxinus* plantation, soil samples were acquired from within the *Larix* row, the *Fraxinus* row, and between the *Larix* and *Fraxinus* rows. After removal of visible plant roots and understory litter, the soil material was sieved through a 2-mm sieve. Aliquots for DNA extraction were stored at −20°C and at 4°C for the complete determination of enzyme activities within a 1-month period. The remaining samples were dried at 65°C to constant weight for the determination of the soil physicochemical properties.

**Table 1:** Site information.

Plantation type		Number	Stand age	Location	Stand density/hm^−2^	Aspect
*Larix* plantation		L-1	28	41°36′0.56″N 125°0′14.63″E	1,025	Southeast
		L-2	28	41°37′45.13″N 125°15′9.39″E	925	
		L-3	28	41°36′59.33″N 125°0′5.61″E	1,050	
*Fraxinus mandshurica* plantation		F-1	24	41°34′27.85″N 124°53′46.43″E	1,225	Southeast
		F-2	24	41°42′49.51″N 125°27′24.32″E	950	
		F-3	24	41°38′29.8″N 124°48′49.1″E	1,350	
*Larix*–*Fraxinus* mixed plantation	M-1	LL-1	28	41°32′9.33″N 125°0′18.9″E	1,212	Southeast
		FF-1	28			
		LF-1	28			
	M-2	LL-2	28	41°45′5.29″N 125°10′7.63″E	1,162	
		FF-2	28			
		LF-2	28			
	M-3	LL-3	28	41°38′10.22″N 125°2′12.13″E	1,237	
		FF-3	28			
		LF-3	28			

L, *Larix* plantation; F, *Fraxinus* plantation; M, *Larix*–*Fraxinus* mixed plantation; LL, *Larix*–*Larix* plantation belt in mixed plantation; FF, *Fraxinus*–*Fraxinus* plantation belt in mixed plantation; and LF, *Larix*–*Fraxinus* plantation belt in mixed plantation.

### Soil Physicochemical Property Analysis

All samples were analyzed for total nitrogen content, nitrate nitrogen content, ammonium nitrogen content, pH, organic matter content, total phosphorus content, available phosphorus content, and moisture content. Analysis of total N content was carried out using an elemental analyzer based on dry combustion (Vario EL III, Germany). Soil organic matter was measured by the external-heat potassium dichromate oxidation method; available nitrogen was measured by the alkaline hydrolysis diffusion method. Total phosphorus was determined by molybdenum antimony resistance colorimetry. Available phosphorus was measured by the NaHCO_3_ extraction method (Yang and Yang, 2020). The moisture content of the soil was determined by drying to a constant weight at 65°C. pH values were determined in deionized water (1:2.5 for soil sample, m/v; Fioretto et al., 2000).

### Soil Enzymes Assays

We examined the activities of five extracellular enzymes involved in soil C, N, and P degradation: β-glucosidase, sucrase, urease, acid phosphatase, and alkaline phosphatase. All soil samples were compared without the matrix, and a soil control was not used in the experiments. Soil enzyme activities were defined as mg of products produced over 24 h per gram of dried sample.

The activity of β-glucosidase was determined using the p-nitrophenol matrix method (Kourtev et al., 2002). Soil sucrase activity was measured by the 3,5-dinitrosalicylic acid method, urease activity was measured by the phenol sodium hypochlorite colorimetric method, acid and alkaline phosphatase activity was estimated using the disodium phenyl phosphate colorimetric method (Ge et al., 2017).

### Total DNA Extraction

In accordance with the manufacturer’s instructions, total microbial community genomic DNA was extracted from 500 mg of soil material using the E.Z.N.A.® soil DNA Kit (Omega Bio-tek, Norcross, GA, United States). There were three duplications for each sample. Duplicate DNA extractions were performed for each sample and pooled. DNA extraction results were tested on 1% agarose gels; DNA concentration and purity were determined with a NanoDrop 2000 UV–Vis spectrophotometer (Thermo Scientific, Wilmington, United States).

### High-Throughput Sequencing of Soil Bacterial 16S rDNA and Fungal ITS Region

The hypervariable V3–V4 region of the bacterial 16S rRNA gene was amplified with the primer pairs 515F (5′-GTGCCAGCMGCCGCGG-3′) and 907R (5’-CCGTCAATT CMTTTRAGTTT-3′) by an ABI GeneAmp® 9,700 polymerase chain reaction (PCR) thermocycler (ABI, CA, United States). The primers ITS3/ITS4 were amplified with the primer pairs ITS3F (5′-GCATCGATGAAGAACGCAGC-3′) and ITS4R (5′-TCCTCCGCTTATTGATATGC-3′). The PCR amplification conditions were as follows: 95°C for 3 min; 27 cycles of 95°C for 30 s, 55°C for 30 s, and 72°C for 45 s; 72°C for 10 min; and indefinite hold at 4°C upon completion. The PCR condition was the same except that the number of fungal PCR cycles was 35. The PCR mixtures contained 2 μl of 10 × TransSTART FastPfu buffer, 2 μl of 2.5 mM dNTPs, 0.8 μl of forward primer (5 μM), 0.8 μl of reverse primer (5 μM), 0.2 μl of TaKaRa rTaq DNA Polymerase, BSA 0.2 μl, 10 ng of template DNA, and sufficient ddH_2_O to achieve a final volume of 20 μl. PCRs were carried out in triplicate. The PCR product was extracted from 2% agarose gel and purified using the AxyPrep DNA Gel Extraction Kit (Axygen Biosciences, Union City, CA, United States), in accordance with the manufacturer’s instructions, and then quantified using a Quantus™ Fluorometer (Promega, United States).

### Bioinformatics Analyses

The original data were allocated to each sample according to a specific barcode, and then, tags and primer sequences were removed. The FLASH software (V1.2.11) was used to splice paired-end reads (Chen et al., 2020). The Qiime software (V1.9.1) was performed to quality control and filter the spliced sequences (Whelan and Surette, 2017). Chimeric sequences were detected and removed by UCHIME (Edgar et al., 2011). Paired-end reads were obtained by MiSeq-sequencing and then spliced on the basis of their overlapping relationships. Quality control and filtering were carried out on the resulting sequences. After samples had been differentiated, operational taxonomic unit (OTU) clustering analysis and species taxonomy analysis were performed; the OTUs were subjected to multiple diversity index analysis and sequencing depth detection. On the basis of taxonomic information, OTUs were included in statistical analysis of community structure at each classification level. To obtain species classification information regarding each OTU, an RDP classifier Bayesian algorithm was used to analyze representative OTU sequences with a 97% similarity level. The bacterial species classification and comparison database were SILVA (Release138)^1^, while the fungal were Unite (Release 8.0).^2^ FAPROTAX database was used to predict the functional potential of bacteria (Sansupa et al., 2021). Functions of fungal communities were classified and analyzed by FUNGuild,^3^ with the fungi divided into pathotrophs, symbiotrophs, and saprotrophs (Nguyen et al., 2016). Each sample’s bacterial and fungal community compositions were determined at all taxonomic levels. Accordingly, various in-depth statistical and visual analyses were performed to determine the community composition and phylogenetic information of multiple samples; these included multivariate analysis and significant difference tests. All the raw datasets in this study were publicly available in the NCBI Sequence Read Achieve (SRA) database with an accession number SRP355360.

### Statistical Analysis

All statistical analyses in this study were performed by SPSS software (SPSS Inc., Chicago, IL). In all analyses, differences were considered statistically significant when *p* < 0.05 and extremely significant when *p* < 0.01. When data did not conform to assumptions of normality, the values were transformed into logarithm and/or square root before analysis. ANOVA of split plot was used to test the effects of microbial diversity (Chao, Shannon, and Simpson index) plantation type, soil depth, and their interaction on soil chemical properties and extracellular enzyme activities (Jones and Nachtsheim, 2009). The plantation types were the main plot, and soil depths were the split plot. Kruskal–Wallis H test was performed to analyze the difference of microbial groups in different soil samples. Additional statistical analyses were conducted using R software. Differences in soil microbial community composition and functions, based on changes in plantation types, were evaluated using principal coordinates analysis (PCoA), and analysis of similarities (ANOSIM) was performed in R using the “vegan” package based on Bray–Curtis dissimilarity distances (Calderón et al., 2017; Otsing et al., 2018). The sequences for all the samples were unified by minimum number of sample sequences before PCoA. BIO-ENV package and distance-based redundancy analysis (db-RDA) were implemented by the vegan package in R software to examine relevance among microbial composition, enzyme activity, and soil nutrient content (Wang et al., 2019; Testa et al., 2021).

## Results

### Soil Physicochemical Properties and Extracellular Enzyme Activities

**Figure 1:**
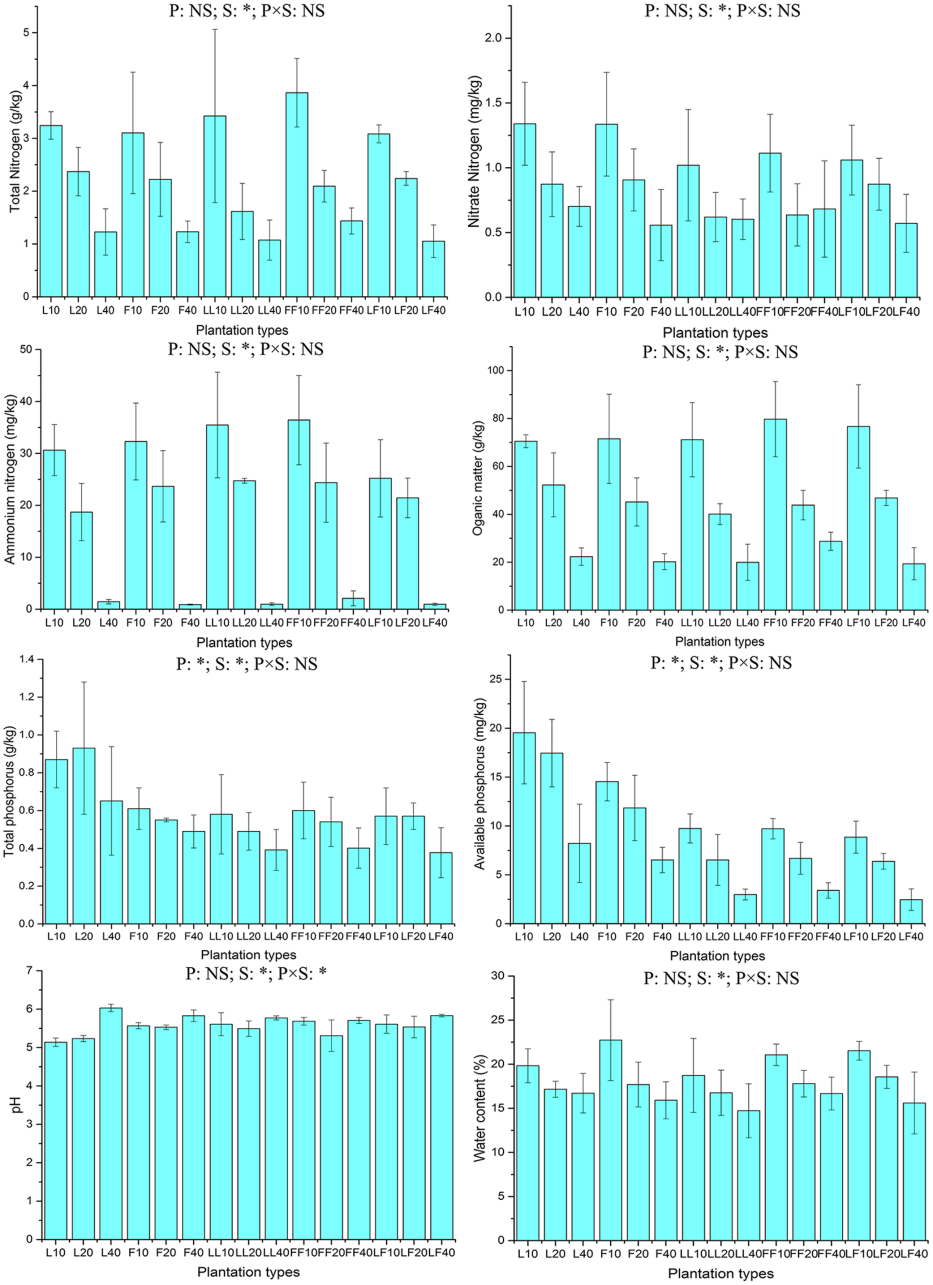
Chemical properties of soil samples. L, *Larix* plantation; F, *Fraxinus* plantation; LL, *Larix*–*Larix* plantation belt in the mixed plantation; FF, *Fraxinus*–*Fraxinus* plantation belt in the mixed plantation; LF, *Larix*–*Fraxinus* plantation belt in the mixed plantation; P, plantation types; S, soil depths; NS, not significant. Error bars show standard errors of the mean (*n* = 3). ANOVA of split plot results are reported. *p < 0.05. Numbers after letters represent soil depths of 0–10, 10–20, 20–40 cm.

ANOVA of split plot showed that the contents of total and available soil phosphorus were significantly affected by plantation type and soil depth (*p* < 0.05; Figure 1). With increasing soil depth, the contents of total and available soil phosphorus gradually decreased (*p* < 0.05). Among the three plantation types, the available soil phosphorus contents were significantly higher in pure *Larix* and *Fraxinus* plantations than in the mixed *Larix*–*Fraxinus* plantation (*p* < 0.05).

Total nitrogen content, nitrate nitrogen content, ammonium nitrogen content, organic matter content, moisture content, and pH in soil were significantly affected by soil depth (*p* < 0.05). With increasing soil depth, the contents of organic matter, total nitrogen, nitrate nitrogen, ammonium nitrogen, and moisture gradually decreased; in contrast, the pH value gradually increased.

**Figure 2:**
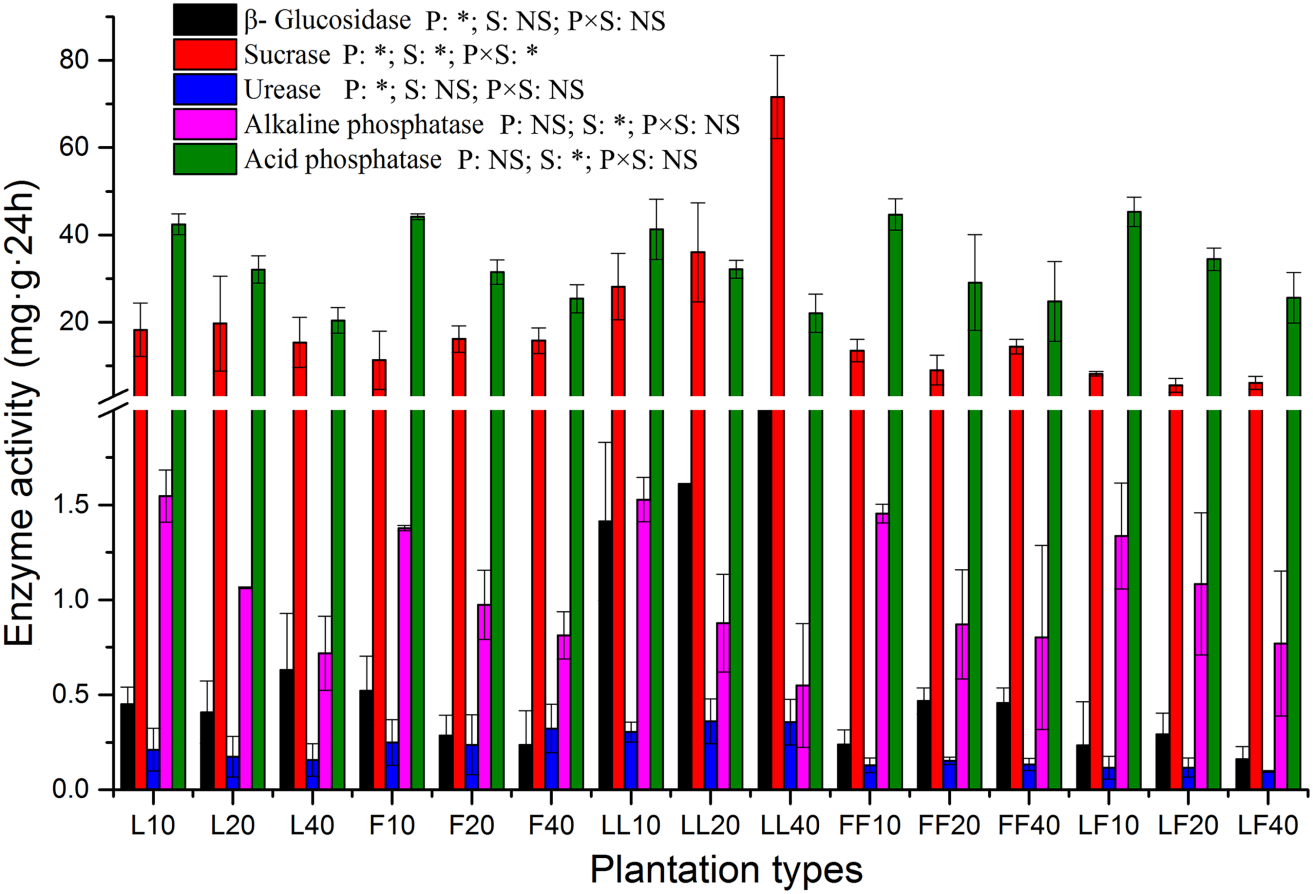
Extracellular enzyme activities in soil samples. Error bars show standard errors of the mean (*n* = 3). ANOVA of split plot results are reported. Refer to Figure 1 legend for relevant abbreviations.

The activities of β-glucosidase, sucrase, and urease were significantly affected by plantation type (*p* < 0.01; Figure 2). Compared with other soil samples, the β-glucosidase activities were significantly improved in the mixed *Larix*–*Larix* plantation soil. The sucrase, alkaline phosphatase, and acid phosphatase activities were significantly affected by soil depth (*p* < 0.01). The alkaline phosphatase and acid phosphatase activities significantly decreased in all three plantation types at increased soil depths. There were significant differences in sucrase activities among plantation types and soil depths (*p* < 0.05).

### Soil Bacterial Community Diversity, Composition, and Function

ANOVA of split plot showed that the indices of Shannon were significantly affected by plantation type and soil depth (*p* < 0.01; Table 2). The index of Simpson was only significantly affected by soil depth (*p* < 0.05). The index of Chao was only significantly affected by plantation type (*p* < 0.05). With the increase in soil depth, bacterial community diversity gradually decreased. Compared with L*arix* plantation, there was higher bacterial community diversity and richness in other plantations.

**Table 2:** Diversity indices of soil bacterial communities in OUT level.

Samples	Shannon	Simpson	Chao
L10	6.15 ± 0.11	0.0073 ± 0.0008	2986.76 ± 24.97
L20	5.99 ± 0.10	0.0078 ± 0.0004	2764.70 ± 114.79
L40	6.02 ± 0.03	0.0074 ± 0.0005	2766.60 ± 93.70
F10	6.34 ± 0.07	0.0068 ± 0.0001	3138.91 ± 43.01
F20	6.20 ± 0.08	0.0071 ± 0.0007	3273.87 ± 135.36
F40	6.16 ± 0.13	0.007 ± 0.0002	3056.30 ± 139.78
FF10	6.23 ± 0.19	0.0069 ± 0.0010	3071.00 ± 162.89
FF20	6.07 ± 0.12	0.0072 ± 0.0009	2938.20 ± 144.98
FF40	5.93 ± 0.06	0.0083 ± 0.0002	2803.00 ± 65.81
LL10	6.26 ± 0.09	0.0069 ± 0.0004	3100.32 ± 231.60
LL20	6.10 ± 0.10	0.0072 ± 0.0007	2866.92 ± 261.20
LL40	6.07 ± 0.02	0.0071 ± 0.0003	2948.14 ± 102.84
LF10	6.29 ± 0.05	0.0064 ± 0.0002	3021.19 ± 123.17
LF20	6.16 ± 0.12	0.0072 ± 0.0002	3179.13 ± 213.48
LF40	6.17 ± 0.17	0.0074 ± 0.0011	3111.91 ± 181.08

Refer to the notes in Table 1 for relevant abbreviations. Numbers after letters represent soil depths of 0–10, 10–20, 20–40 cm.

**Figure 3:**
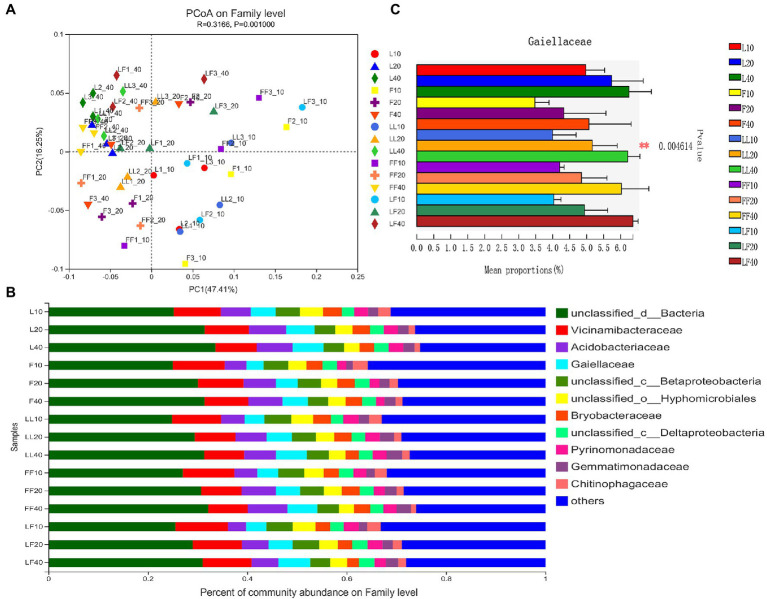
Bacterial community composition in different soil samples. **(A)** Principal coordinates analysis of bacterial community composition. **(B)** Bacterial community structure at the level of family. **(C)** The results of Kruskal–Wallis H test on the difference of *Gaiellaceae* in different soil samples. Refer to Figure 1 legend for relevant abbreviations.

Principal coordinates analysis showed significant differences in bacterial community composition at family level among soil depth (ANOSIM, *R* = 0.32, *p* = 0.001; Figure 3A). There was no significant difference in bacterial community composition among plantation types (ANOSIM, *R*^2^ = 0.15, *p* = 0.059). In total, 12 bacterial families were identified with the relative abundances >3% (Figure 3B). Among them, *Gaiellaceae* and *Acidobacteriales* were dominant flora. With increasing soil depths, the relative abundance of *Gaiellaceae* and *Acidobacteriales* was gradually increased (Figure 3C). Especially, the relative abundance of *Gaiellaceae* significantly increased in all soil samples (*p* < 0.05). Under the same soil depth, there was no significant difference in bacterial community composition among different plantation types.

**Figure 4:**
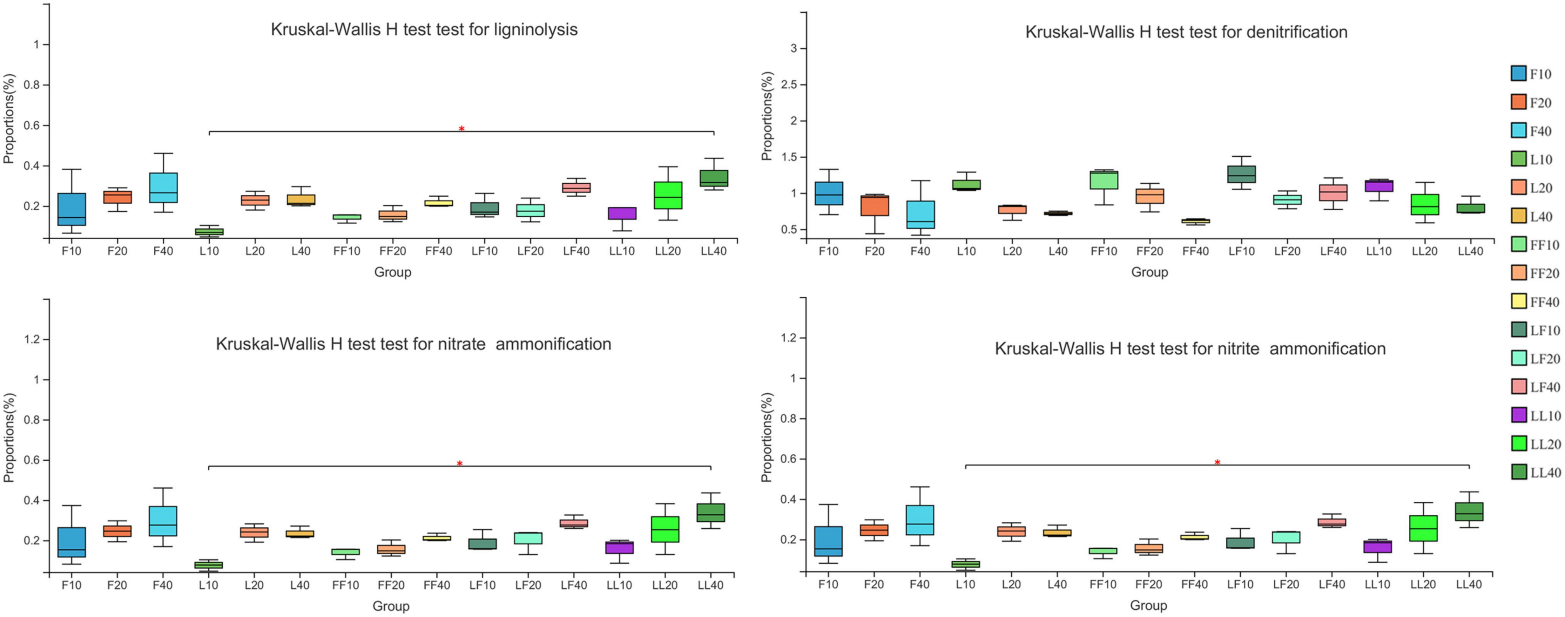
Functional bacterial community categories involved in nutrient cycling function. Error bars show standard errors of the mean (*n* = 3). Refer to Figure 1 legend for relevant abbreviations.

Bacterial functional categories inferred by FAPROTAX were significantly differed among different soil depths (Figure 4). ANOVA of split plot showed that the relative abundances of functional genes for bacterial ligninolysis, nitrate ammonification, and nitrite ammonification were significantly affected by soil depth (*p* < 0.01). Kruskal–Wallis H test showed that with increasing soil depth, bacterial ligninolysis, nitrate ammonification, and nitrite ammonification functional genes significantly increased (*p* < 0.05). On the contrary, bacterial denitrification functional genes gradually decreased in different soil samples. There were no significant differences in bacterial metabolism functions in the same soil layer among the three plantation types (*p* > 0.05).

### Soil Fungal Community Diversity, Composition, and Function

ANOVA of split plot showed that the indices of Chao were significantly affected by plantation type and soil depth (*p* < 0.05; Table 3). The index of Shannon and Simpson was not significantly affected by plantation type and soil depth (*p* > 0.05). Compared with *Larix* plantation, there was higher fungal community richness in other plantations. With the increase in soil depth, fungal community richness gradually decreased in all plantation types.

**Table 3:** Diversity indices of soil fungal communities in OUT level.

Sample	Shannon	Simpson	Chao
L10	3.90 ± 0.26	0.06 ± 0.01	656.94 ± 169.54
L20	3.75 ± 0.29	0.06 ± 0.02	480.73 ± 100.32
L40	4.05 ± 0.26	0.05 ± 0.03	425.61 ± 65.18
F10	3.92 ± 1.03	0.14 ± 0.15	982.26 ± 46.34
F20	4.42 ± 0.23	0.04 ± 0.01	732.68 ± 119.73
F40	4.62 ± 0.13	0.03 ± 0.01	654.12 ± 54.76
FF10	4.39 ± 0.48	0.06 ± 0.02	1021.13 ± 154.91
FF20	3.62 ± 1.46	0.16 ± 0.21	703.07 ± 40.55
FF40	4.03 ± 0.30	0.05 ± 0.02	520.34 ± 71.98
LL10	3.85 ± 1.19	0.11 ± 0.13	907.37 ± 84.21
LL20	3.69 ± 1.26	0.15 ± 0.18	722.13 ± 49.87
LL40	3.59 ± 1.34	0.16 ± 0.20	568.35 ± 99.72
LF10	3.97 ± 1.02	0.12 ± 0.15	934.56 ± 126.02
LF20	4.05 ± 0.61	0.07 ± 0.05	822.62 ± 36.78
LF40	3.92 ± 0.79	0.10 ± 0.08	720.03 ± 179.04

Refer to the notes in Table 1 for relevant abbreviations. Numbers after letters represent soil depths of 0–10, 10–20, 20–40 cm.

**Figure 5:**
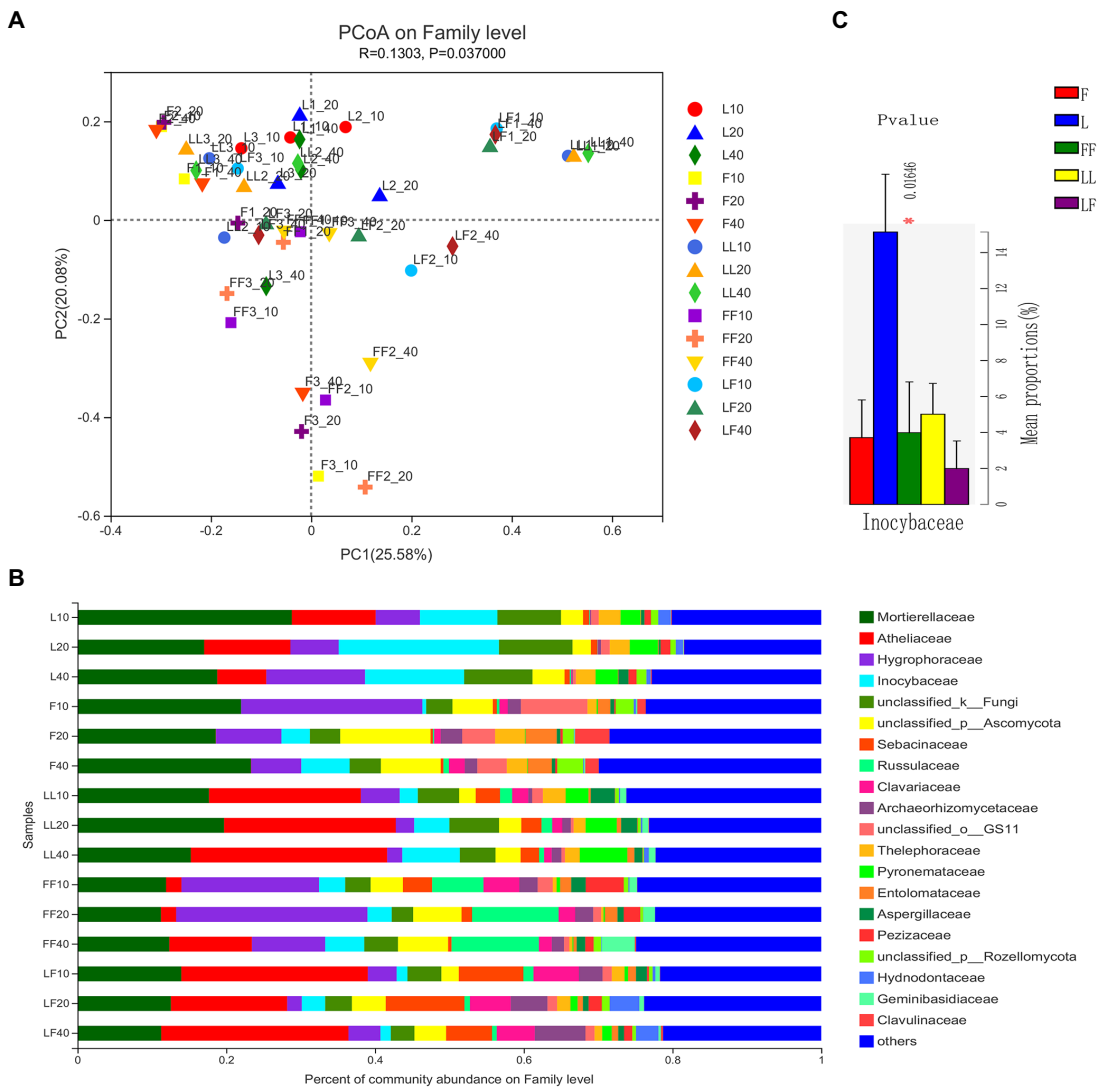
Fungal community composition in different soil samples. **(A)** Principal coordinates analysis of fungal community composition. **(B)** Fungal community structure at the level of family. **(C)** The results of Kruskal–Wallis H test on the difference of *Inocybaceae* in different soil samples. Refer to Figure 1 legend for relevant abbreviations.

Principal coordinates analysis showed significant differences in fungal community composition at family level among plantation types (ANOSIM, *R* = 0.13, *p* = 0.037; Figure 5A). There were significant differences in fungal community composition among plantation types (ANOSIM, *R*^2^ = 0.29, *p* = 0.001). In total, 20 fungal families were identified with the relative abundances >3% (Figure 5B). *Mortierellaceae*, *Atheliaceae*, *Hygrophoraceae*, and *Inocybaceae* were dominant flora in all soil samples. Compared with other plantations, the relative abundance of *Inocybaceae* was significantly higher in the *Larix* plantation (*p* < 0.05; Figure 5C). The relative abundances of *Atheliaceae* were lower in *Fraxinus* soils than other types of plantation soils. There were no significant differences in fungal community composition with increasing soil depths in the same type of plantation.

**Figure 6:**
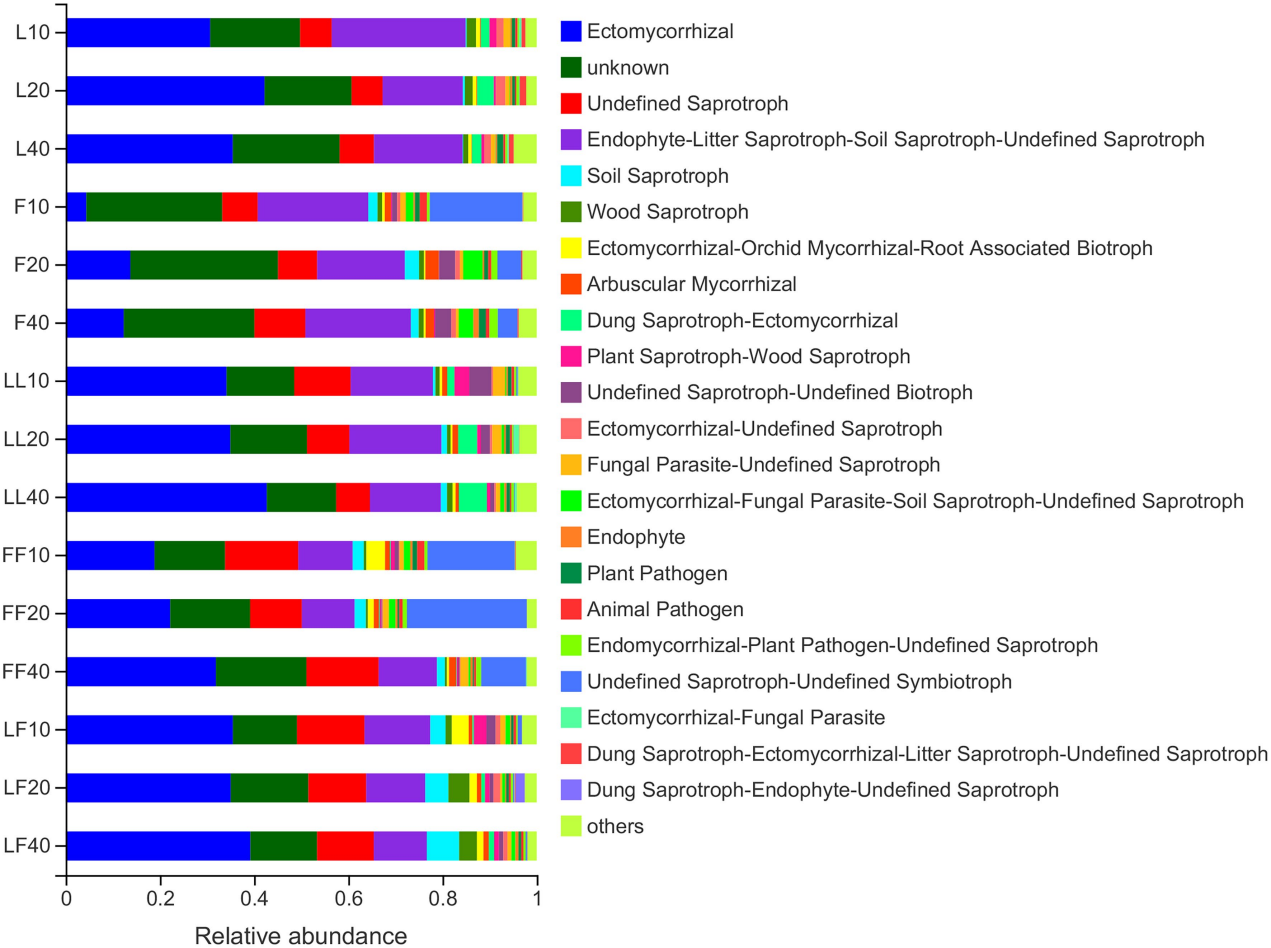
Variations in fungal functional group compositions inferred by FUNGuild. Refer to Figure 1 legend for relevant abbreviations.

Fungal functions were classified by FUNGuild (Figure 6). The results showed that the mean relative abundance of guild-undefined saprotrophs comprised approximately 48% of all fungi OTUs. The mean relative abundance of Ectomycorrhizae comprised approximately 28% of all fungi OTUs. The relative abundances of saprotrophs were significantly higher in *Fraxinus* and *Fraxinus*–*Fraxinus* plantation soils than in other plantation soils (*p* < 0.05). In contrast, the relative abundance of Ectomycorrhizae was significantly lower in *Fraxinus* plantation soil than in other plantation soils (*p* < 0.05).

### Correlation Analysis of Environmental Factors

**Figure 7:**
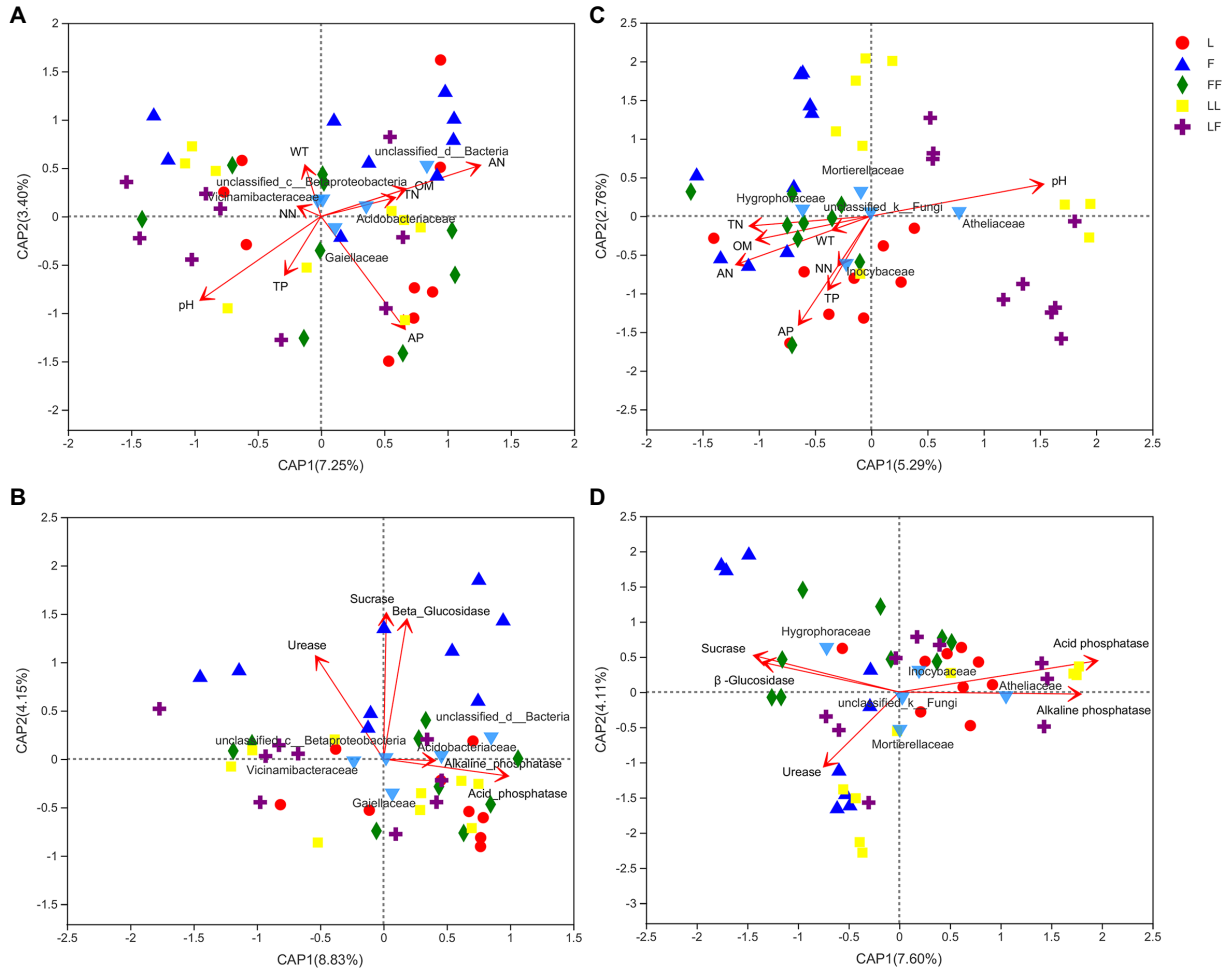
Distance-based redundancy analysis (db-RDA) to show correlation between microbial community and environmental factors in different soil samples. **(A)** Bacterial families and nutrient; **(B)** bacterial families and enzymatic activity; **(C)** fungal families and nutrient; **(D)** fungal families and enzymatic activity. TN, total nitrogen; AN, ammonium nitrogen; NN, nitrate nitrogen; TP, total phosphorus; AP, available phosphorus; OM, organic matter; WT, water content. Refer to Figure 1 legend for relevant abbreviations.

db-RDA showed that bacterial community compositions at family level were significantly correlated with pH, ammonium nitrogen, and available phosphorus contents (*R*^2^ = 0.18, *p* = 0.017; *R*^2^ = 0.18, *p* = 0.019; *R*^2^ = 0.17, *p* = 0.02; Figure 7A). Among the bacterial families, *Gaiellaceae* and *Acidobacteriales* were positively correlated with available phosphorus and ammonium nitrogen contents, while negatively correlated with pH alone. Fungal community compositions were significantly correlated with pH, ammonium nitrogen, total nitrogen, available phosphorus, organic matter, and total phosphorus contents (*R*^2^ = 0.44, *p* = 0.001; *R*^2^ = 0.33, *p* = 0.001; *R*^2^ = 0.20, *p* = 0.01; *R*^2^ = 0.40, *p* = 0.001; *R*^2^ = 0.20, *p* = 0.008; *R*^2^ = 0.17, *p* = 0.017; Figure 7C). Among the fungal families, *Inocybaceae* was positively correlated with available phosphorus, ammonium nitrogen, and total phosphorus contents and negatively correlated with pH. *Atheliaceae* was opposite to *Inocybaceae*.

Bacterial community compositions were significantly correlated with acid phosphatase, β-glucosidase, sucrase, and urease activities (*R*^2^ = 0.18, *p* = 0.013; *R*^2^ = 0.58, *p* = 0.001; *R*^2^ = 0.61, *p* = 0.001; *R*^2^ = 0.32, *p* = 0.001; Figure 7B). Among the bacterial families, *Gaiellaceae* and *Acidobacteriales* were positively correlated with acid phosphatase and alkaline phosphatase activities. Fungal community compositions were significantly correlated with β-glucosidase, sucrase, acid phosphatase, alkaline phosphatase, and urease activity (*R*^2^ = 0.26, *p* = 0.007; *R*^2^ = 0.30, *p* = 0.004; *R*^2^ = 0.53, *p* = 0.001; *R*^2^ = 0.42, *p* = 0.001; *R*^2^ = 0.19, *p* = 0.018; Figure 7D). Among the fungal groups, *Inocybaceae* was positively correlated with acid phosphatase and alkaline phosphatase activities, while negatively correlated with β-glucosidase, sucrase, and urease activities.

## Discussion

### Effects of Mixed Plantations on Soil Bacterial and Fungal Communities

It was found that vegetation type and soil depth had significant effects on microbial community diversity. In this study, there was lower microbial community diversity and richness in *Larix* plantation than other plantations. The increase of microbial community biodiversity was conducive to soil health and forest growth (Van Bruggen and Semenov, 2000). Higher microbial diversity can increase the number and resilience of plant beneficial functions, which enable plants to produce beneficial characteristics that are difficult to obtain from any isolated species (Saleem et al., 2019). Therefore, cultivating coniferous and broad-leaved mixed plantation has the potential advantage of improving forest productivity. With the increase in soil depth, bacterial community diversity and fungal richness were gradually decreased. These findings consist with the previous study (Too et al., 2018). The possible reason for the results was that surface soil was rich in nutrients under the action of soil leaching and forest litter decomposition, which could provide niche for the growth of more microbial groups.

Bacterial communities and functions were shaped by soil depth but not plantation type. It is widely accepted that mixed plantations exhibit significant effects on soil microbial community structure (Chen et al., 2013; Pereira et al., 2017), although this effect might depend on soil depth. For example, Zhang et al. (2021) showed that mixed plantations did not significantly alter bacterial community structure in the upper soil. In this study, ANOSIM analysis revealed that there were significant differences in bacterial community composition among different soil depths rather plantation type. One possible reason was that the composition of soil bacterial community was mainly driven by soil nutrients (Dong et al., 2021). In this study, except phosphorus, other nutrient content had no significant differences among plantation types. Compared with other soil environmental factors such as pH, organic C, and inorganic N, phosphorus has a relatively low weight in shaping bacterial community structure (Hu et al., 2014; Wang et al., 2018). With the increasing soil depth, the nutrient content and pH significantly changed. This led to significant changes in bacterial community composition. These results were in agreement with the research of Zhang et al. (2016) and Li et al. (2019); the changes of total carbon, total nitrogen, and pH in different soil depths led to significant differences in bacterial community composition. Huang et al. (2013) showed that soil edaphic characteristics and soil profile might shape bacterial community functions; metabolic profiles revealed the carbon source utilization capacity in the surface layer. Using the BIOLOG system, Griffiths et al. (2003) and Braun et al. (2006) showed that the bacterial capability for utilizing diverse substrates decreased with soil depth. Our study showed that except for *Larix* plantation, ligninolysis, nitrate ammonification, and nitrite ammonification functional gene abundances significantly decreased with increasing soil depth in other plantations. This suggests that soil depth, rather than plantation type, significantly affected bacterial community structure and functional potential of nutrient cycling.

The abundance and diversity of fungal communities are closely related to tree species composition, and microbial communities associated with mixed species are more active (Khlifa et al., 2017). Notably, plant species, phylogenetic relationships, and plant traits affect the compositions of rhizosphere fungal communities (Sweeney et al., 2020). Our results suggested that soil fungal community composition significantly differed among the three plantation types. The main soil fungal families were *Atheliaceae*, *Hygrophoraceae*, and *Inocybaceae*, which belonged to the phylum of Basidiomycota. Most Basidiomycota were soil saprotrophs, and the relative abundances of soil saprotrophs were significantly higher in *Fraxinus* and *Fraxinus*–*Fraxinus* plantations than in other plantations. One possible explanation is that *Fraxinus* litter contains more nutrients than *Larix* litter, thus providing more niches for soil Basidiomycota. This was confirmed by Wu D. et al. (2019a), who showed that saprophytic fungi were more abundant in plantations with high soil nutrients. The mixed *Larix* and *F. mandshurica* plantation also had a higher relative abundance of ectomycorrhizal fungi than did a pure *F. mandshurica* plantation. Ectomycorrhizal fungi commonly occur in conifer roots, and the mixture of broadleaf trees with conifers would likely increase ectomycorrhizal fungal diversity (Ishida et al., 2007). Wu W. et al. (2019b) showed that mixed plantations of *F. mandshurica* and *P. koraiensis* contained a highly diverse and abundant population of ectomycorrhizal fungi. In that study, most *Inocybaceae* were ectomycorrhizal fungi (Matheny et al., 2009), and the relative abundance of *Inocybaceae* was significantly higher in *Larix* plantation soil. Our study indicated that mixed *Larix* and *F. mandshurica* plantations had significant effects on the fungal community. Thus, there is a need to closely monitor the impacts of mixed plantations on fungal communities in future sustainable management efforts.

### Relationships Among Microbial Community Composition, Enzyme Activity, and Nutrient Content

This study found that mixed plantations did not significantly change general soil fertility or organic C and total N contents, compared with monoculture plantations. These results were consistent with the findings of previous studies, in which no significant differences were observed in soil organic carbon, total nitrogen, and available nitrogen contents among the three plantation types (Zhang et al., 2021). However, our study showed that plantation types and soil depths significantly affected the total soil phosphorus and available phosphorus contents. Among the three plantation types, the available soil phosphorus contents were significantly higher in *Larix* and *Fraxinus* plantations than in the mixed *Larix*–*Fraxinus* plantation. With increasing soil depth, available phosphorus content significantly decreased in all three plantation types. In contrast, some studies found significant increases in available phosphorus contents in the mixed-species treatments (Rachid et al., 2013; Zhang et al., 2021). One explanation is that faster growth leads to decreases in available phosphorus contents in mixed *Larix*–*Fraxinus* plantations (Feng et al., 2021).

Changes in soil microbial community structure serve as important indicators of soil quality and soil changes (Sharma et al., 2010). Bacteria and fungi reportedly contribute to nutrient bioavailability in degraded soils (Rashid et al., 2016). Dang et al. (2018) showed that soil organic carbon, total nitrogen, and available phosphorus contents were significantly correlated with soil bacterial communities; soil organic carbon, total nitrogen, total phosphorus, available phosphorus, and nitrate nitrogen contents were significantly correlated with soil fungal communities. The present study showed that bacterial and fungal community compositions were significantly correlated with the available phosphorus content. Our results showed that available phosphorus contents were significantly higher in *Larix* plantations than in the mixed *Larix*–*Fraxinus* plantation. Furthermore, higher relative abundances of *Inocybaceae* were observed in *Larix* plantation soils. Some fungi in *Inocybaceae* family have higher phosphate-solubilizing activity; thus, they can dissolve nearly all types of phosphorus in soil (Carrino-Kyker et al., 2016). This indicated that the higher abundances of *Inocybaceae* might increase inorganic phosphorus contents in *Larix* plantation soils.

Spatial and temporal variations in soil microorganisms are affected by differences in dominant tree species; these can ultimately change the availability and dynamics of soil nutrients, as well as shifts in microbial community composition, during adaptation to new environmental conditions. Physicochemical changes along the soil profile may be the main factor that controls the properties of bacterial communities (Ranjard and Richaume, 2001; Fierer et al., 2003). In the soil profile, total phosphorus content, available phosphorus content, pH value, organic matter content, and moisture content were significantly correlated with soil depth; these findings were consistent with the results of previous studies (Blume et al., 2002; Griffiths et al., 2003; Eilers et al., 2012). In the present study, the abundance of *Gaiellaceae* was positively correlated with available phosphorus content and phosphatase activity. It is reported that the members of family *Gaiellaceae* had high alkaline and acid phosphatase producing activity (Albuquerque and Costa, 2014; López-Lozano et al., 2020). With increasing soil depth, the relative abundance of *Gaiellaceae* significantly increased. It may be the distribution and physiological characteristic of *Gaillaceae* that makes the content of soil available phosphorus decrease with the increase in soil depth.

Soil enzyme activity plays a vital role in the turnover of carbon, nitrogen, and phosphorus; it is closely related to the distribution of soil microbial communities (Midgley and Phillips, 2016; Xu et al., 2017). Our results indicated that plantation type and soil depth had distinct effects on enzyme activities. In general, broad-leaved forests and mixed coniferous broad-leaved forests have higher soil enzyme activities than do coniferous forests alone (Hu et al., 2006; Xing et al., 2009). Similar to the previous results, our study showed significantly increased sucrase and β-glucosidase activities in *Larix*–*Larix* plantation soil. Alkaline phosphatase and acid phosphatase activities were significantly decreased with soil depth in the three plantation types, which is consistent with findings by Samuel et al. (2010). Distance-based redundancy analysis revealed that *Gaiellaceae* and *Inocybaceae* abundances were positively correlated with available phosphorus content and phosphatase activity. This indicated that soil available phosphorus variations were closely related to the relative abundance of *Gaiellaceae* at different soil depths and to the relative abundance of *Inocybaceae* in different plantation types. In general, our study indicated that mixed *Larix*–*Fraxinus* plantations resulted in distinct microbial groups, relative to the respective monocultures; these groups had positive effects on soil phosphorus content, potentially increasing the need for phosphorus anthropogenic fertilization.

## Conclusion

This study characterized microbial communities and nutrient cycling function in different plantation type and soil depth. Notably, available soil phosphorus was significantly affected by plantation type and soil depth. The mixed *Larix*–*Fraxinus* plantation had a significant effect on soil fungal community structure and nutrient cycling function. In contrast, bacterial community characteristics were closely related to soil depth. Variations in soil available phosphorus among plantation types and soil depths were correlated with the relative abundances of *Inocybaceae* and *Gaiellaceae* respectively. These findings provided novel insights into the relationships among soil microbial communities, enzyme activities, and nutrient contents according to plantation type and soil depth. There is a need for closer assessment of changes in fungal communities during future efforts to achieve sustainable management of mixed plantations.

## Data Availability Statement

The datasets presented in this study can be found in online repositories. The name of the repository and accession numbers can be found at: SRA, NCBI; accession: SRP355360; bioproject: PRJNA797909.

## Author Contributions

WW and JF performed conceptualization. WW did methodology, software, writing—original draft preparation, and visualization. WW, JW, and JF done validation. RB performed formal analysis. WW, JW, QW, and JF contributed to investigation. JF contributed to re-sources, funding acquisition, and data curation. JW, QW, and JF were involved in writing—review and editing. SY, PB, ZW, and DC supervised the study. All authors contributed to the article and approved the submitted version.

## Funding

This research was financially supported by National Key Research Program of China (2017YFD060040103), the Shandong Province Double Hundred Talent Plan (WSG20200001), and Doctoral Fund Project of Jinan University (XBS2103).

## Conflict of Interest

The authors declare that the research was conducted in the absence of any commercial or financial relationships that could be construed as a potential conflict of interest.

## Publisher’s Note

All claims expressed in this article are solely those of the authors and do not necessarily represent those of their affiliated organizations, or those of the publisher, the editors and the reviewers. Any product that may be evaluated in this article, or claim that may be made by its manufacturer, is not guaranteed or endorsed by the publisher.

## Footnotes

^1^ http://www.arb-silva.de

^2^ http://unite.ut.ee/index.php

^3^ https://github.com/UMNFuN/FUNGuild
